# Trichoblastomas Mimicking Basal Cell Carcinoma: The Importance of Identification and Differentiation

**DOI:** 10.7759/cureus.8272

**Published:** 2020-05-25

**Authors:** Parth Patel, Shiri Nawrocki, Kelsey Hinther, Amor Khachemoune

**Affiliations:** 1 Dermatology, Montefiore Medical Center, Albert Einstein College of Medicine, New York, USA; 2 Dermatology, Rutgers-Robert Wood Johnson Medical School, Piscataway, USA; 3 Dermatology, University of Saskatchewan, Saskatoon, CAN; 4 Dermatology, State University of New York Downstate Medical Center, Brooklyn, USA

**Keywords:** trichoblastoma, basal cell carcinoma, follicular germinative cells

## Abstract

Trichoblastoma is a rare, slow-growing, benign cutaneous tumor derived from follicular germinative cells. Trichoblastoma commonly appears as an asymptomatic, symmetrical, well-circumscribed, skin-colored to brown or blue-black papule or nodule. It may appear clinically and histologically similar to basal cell carcinoma, making its diagnosis challenging. Even on dermoscopy, it is challenging to differentiate trichoblastoma from basal cell carcinoma. In practice, it is important to differentiate the two, because the choice of treatment and resulting prognosis differ between the lesions. Surgical biopsy to analyze histopathological and immunohistochemical differences is the gold standard for diagnosing and differentiating trichoblastoma from basal cell carcinoma. Trichoblastoma typically has a favorable prognosis, with a low incidence of recurrence, progression or association with malignancy. This paper provides a review of the epidemiology, clinical presentation, dermoscopy, histology, immunochemistry, treatment, and prognosis of trichoblastoma.

## Introduction and background

Trichoblastoma is a rare, slow-growing, benign cutaneous tumor derived from follicular germinative cells [1-3]. Headington first illustrated trichoblastoma in 1970 as a follicular differentiated neoplasm [4]. In 1993, Ackerman et al. further described trichoblastoma to include all follicular germinative cell-derived benign cutaneous tumors [5,6].

The main differential diagnosis for trichoblastoma is basal cell carcinoma, since the two resemble one another both clinically and dermoscopically [2,3]. Basal cell carcinoma, first described in 1827 by Jacob, is the most common malignancy worldwide, accounting for 75% of all cutaneous malignancies [7,8]. Basal cell carcinoma is derived from basal cells of the epidermis, and rare subtypes such as infundibulocystic basal cell carcinoma originate from hair follicle-derived cells [6]. While basal cell carcinoma rarely metastasizes, it is locally invasive [9]. In contrast, trichoblastoma rarely undergoes a malignant transformation into trichoblastic carcinoma, which is a locally invasive and metastatic tumor [1].

The main objective of this paper is to review the epidemiology, clinical presentation, dermoscopic findings, histology, immunochemistry, treatment, and prognosis of trichoblastoma.

## Review

Methods

An extensive literature review of all articles (written in English) published from inception through August 15, 2019, was completed using the following: PubMed, Google Scholar, and the Cochrane Library. The search terms included “trichoblastoma,” “basal cell carcinoma,” “Mohs surgery,” “skin adnexal tumors/neoplasms,” and “follicular tumors.”

Epidemiology

Trichoblastoma is a rare tumor for which the exact incidence and prevalence are not known. Trichoblastoma can arise at any age; however, it is more common among adults between the ages of 40 and 50 years [3]. No race predominance has been reported. Similarly, basal cell carcinoma also commonly arises in adults after the fourth decade of life [8]. The incidence in males is approximately 30% higher than that in females [10]. Basal cell carcinoma is especially common in lighter skin tones, occurring very rarely in darker skin tones. In the United States, the lifetime risk of developing basal cell carcinoma in the white population is approximately 30% [10-14].

Clinical presentation

Generally, trichoblastoma appears as an asymptomatic, well-circumscribed, symmetrical, smooth bordered, skin-colored to brown or blue-black papule or nodule with predominance on the head and neck, with a predilection reported to occur on the scalp [1,2,15]. The overlying epidermis is alopecic, thin, and hyperpigmented. Although it commonly appears as an isolated lesion, multiple lesions have been reported. It may have superficial telangiectasias and appear ulcerated. Most of the lesions are less than 2 cm in diameter but range from 5 mm to 8 cm in diameter [16-20]. Rarely, trichoblastoma has been reported to present on the proximal extremities, trunk, and perianal and genitals areas [21].

The clinical appearance of basal cell carcinoma is variable. It most commonly presents as a pearly, pinkish nodule with overlying telangiectasias and a rolled border, in sun-exposed areas [8]. Various basal cell carcinoma subtypes exist based on histological features and include nodular, nodulocystic, micronodular, superficial, infiltrative, morpheaform, and pigmented types. Approximately 70% of the basal cell carcinomas occur on the face, exemplifying the role of sun-exposure in basal cell carcinoma formation, while 15% arise on the trunk. Basal cell carcinoma is rarely found in sun-protected areas like the perianal skin, vulva, or penis [22].

Pathophysiology

The pathophysiology of trichoblastoma is poorly understood [17,23]. They are associated with certain genetic conditions or syndromes (familial trichoblastoma), including the Brooke-Spiegler disease and the Brooke­-Fordyce syndrome. However, most trichoblastoma cases are isolated and sporadic. Some sporadic and solitary tumors are associated with certain genetic mutations, specifically 9q22.3. In contrast, the pathophysiology of basal cell carcinoma is attributed to environmental, phenotypic, and genetic factors. Aside from ultraviolet radiation exposure, which is the most important risk factor for basal cell carcinoma, other established risk factors include radiation therapy, chronic immunosuppressive therapy, long-term arsenic exposure, nevoid basal cell carcinoma syndrome, and Gorlin syndrome. Mutations in various proto-oncogenes and tumour-suppressive genes, as well as hedgehog signaling pathway malfunction, have been found to be associated with the development of basal cell carcinoma [24,25]. Vismodegib and sonidegib have been approved as hedgehog pathway inhibitors, and itraconazole is used off-label as a hedgehog pathway inhibitor, for the treatment of advanced or metastatic basal cell carcinoma [26]. In a recent meta-analysis, Xie and Lefrançois showed the efficacy of vismodegib, sonidegib, and itraconazole to be 61.9%, 55.2%, and 50%, respectively [27].

Diagnosis

Histopathology

Clinically, trichoblastoma and basal cell carcinoma may be difficult to differentiate, especially between the large nodular variant of trichoblastoma and the nodular variant of basal cell carcinoma. Even on dermoscopy, it is a challenge to differentiate trichoblastoma from basal cell carcinoma. One small, but the possible differentiating factor, as noted by Ghigliotti et al., is the presence of significantly more blue-grey ovoid nests and blue-grey globules in basal cell carcinoma than in trichoblastoma; however, some level of these findings are present in both lesions [3]. In this respect, a surgical biopsy to examine histopathological and immunohistochemical differences remains the gold standard for diagnosing trichoblastoma and basal cell carcinoma (Table 1) [20,28]. Dermatopathologists are aware of the diagnosis of trichoblastoma; however, this is not commonly encountered by general pathologists and the features may be misinterpreted.

**Table 1:** Histological differentiation between trichoblastoma and basal cell carcinoma ^1^The connection may not always be visualized secondary to tangential sectioning of the specimen

Trichoblastoma	Basal cell carcinoma
Absent/focal mitosis	Mitosis
Absent/focal necrosis	Variable necrosis
No inflammatory infiltrate	Lymphocytic infiltrate
No connection with epidermis	Connection with epidermis^1^
Myxoid stromal induction	Variable mucinous stroma

Histologically, trichoblastoma is a well-circumscribed, symmetrical, and basaloid tumor with no epidermal connections. It is usually located in the mid to lower dermis and generally does not invade the subcutaneous tissue. The tumor is composed of irregular nests of basaloid cells, resembling basal cell carcinoma. Trichblastoma differs from basal cell carcinoma with its variable stromal condensation and pilar differentiation [20,29,30]. Additionally, basal cell carcinoma is a basaloid neoplasm that originates from the epidermis. As opposed to trichoblastoma, it is characterized by basaloid nodules, with prominent peripheral palisading and clefting between the neoplasm and surrounding stroma.

In 1993, Ackerman proposed that a trichoblastoma encompasses all adnexal tumors of follicular germinative cells, which demonstrate benign features, such as smooth symmetric growth patterns, sharp demarcation, and smooth borders [5,6]. Trichoblastoma was further classified into seven subtypes: small nodular, large nodular, retiform (giant solitary trichoepithelioma), cribriform (classic trichoepithelioma), racemiform (non-classic trichoepithelioma), columnar (desmoplastic trichoepithelioma), and adamantinoid (cutaneous lymphadenoma) [1,15,20]. The presence of follicular germinative (basaloid) cells is a common histopathological characteristic among all the subtypes [30,31].

Nodular Trichoblastoma

**Figure 1:**
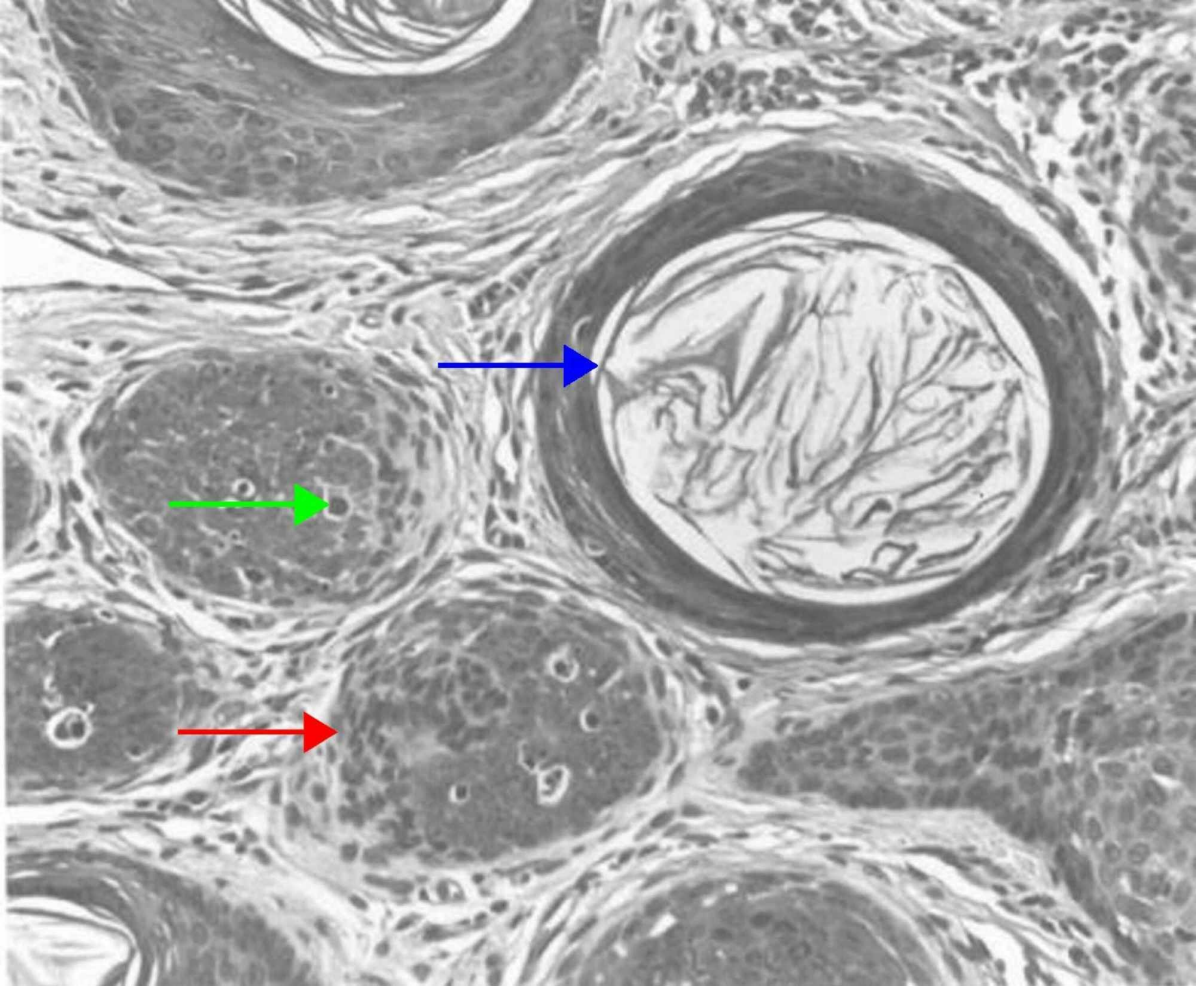
Nodular (large) trichoblastoma The large nodules are crowded and mostly solid (red arrow), but some are punctuated by an infundibulocystic structure that contains corneocytes (blue arrow). Follicular germlike structures are wedded often to a follicular papilla. Some neoplastic cells are necrotic, as evidence by pyknosis and karyorrhexis (green arrow). (Adapted with permission from Ackerman A, Reddy V, Soyer H. Trichoblastoma. In: Ackerman A, Reddy V, Soyer H, editors. Neoplasms with Follicular Differentiation. New York: Ardor Scribendi; 2001. p. 415. Copyright Ardor Scribendi. All rights reserved)

The nodular variant presents as a solitary, skin-colored nodule or papule in the head and neck region of adults [20]. Large nodules are more common than small nodules. The small nodular subtype of trichoblastoma is characterized by small aggregates that resemble a follicular germ in association with a follicular papilla, and a fibrocyte-rich stroma. In the large nodular subtype, the aggregates of follicular germinative cells are both larger in size and fewer in number than in the small nodular subtype (Figure 1) [5,6]. Both variants are well-circumscribed tumors that involve the entire reticular dermis, and usually extend into the upper portion of the subcutis. In certain cases, the small nodular subtype may extend as far as the skeletal muscle, but typically does not impinge upon the muscle fibers [31].

Retiform Trichoblastoma

**Figure 2:**
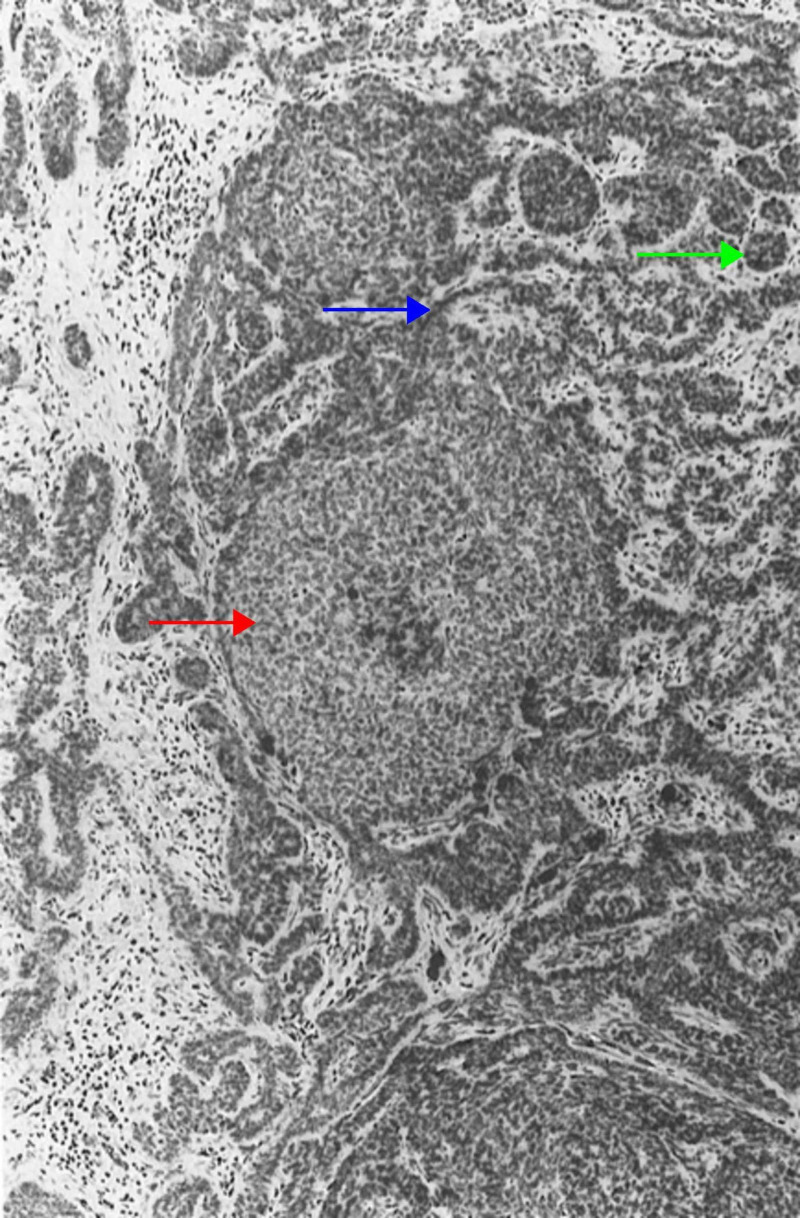
Retiform trichoblastoma Follicular germinative cells arranged in nodules (red arrow). A second population of neoplastic cells, presumably modified germinative ones, is arrayed in reticulations (blue arrow). At the periphery of cords of reticulations are attempts at formation of follicular germs (green arrow). (Adapted with permission from Ackerman A, Reddy V, Soyer H. Trichoblastoma. In: Ackerman A, Reddy V, Soyer H, editors. Neoplasms with Follicular Differentiation. New York: Ardor Scribendi; 2001. p. 524. Copyright Ardor Scribendi. All rights reserved)

Retiform trichoblastoma is a large tumor located in the dermis or subcutaneous tissue, histologically characterized by long cords and columns of follicular germinative cells anastomosing to form a netlike pattern (Figure 2) [5,6]. Other histological trichoblastoma variants, including the cribriform and racemiform types, may be seen in these tumors [19,30,32].

Giant solitary trichoepithelioma is a type of retiform trichoblastoma. The lesion is several centimeters in diameter and is most commonly located on the thigh or perianal region in older adults [33,34].

Cribriform Trichoblastoma

**Figure 3:**
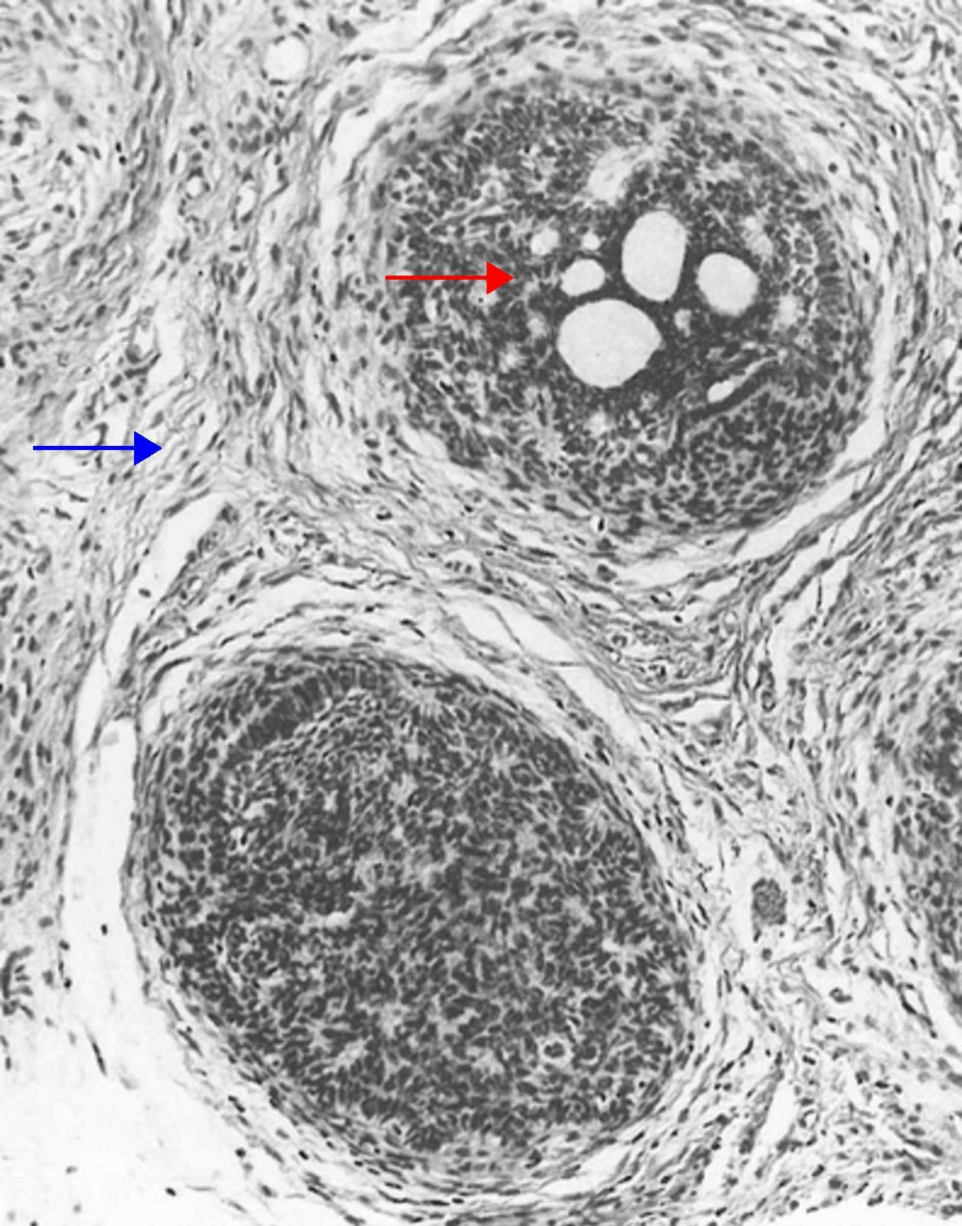
Cribriform (trichoepithelioma) trichoblastoma Fibroepithelial units separated from one another by clefts. The epithelial component is made up of follicular germinative cells arranged in a sieve-like pattern (red arrow), and the nonepithelial component is composed of fibrous tissue that is richly fibrocystic and resembles perifollicular sheath (blue arrow). The germs are in contiguity with a follicular papilla. Some aggregations are continuous with a preexisting infundibulum. (Adapted with permission from Ackerman A, Reddy V, Soyer H. Trichoblastoma. In: Ackerman A, Reddy V, Soyer H, editors. Neoplasms with Follicular Differentiation. New York: Ardor Scribendi; 2001. p. 535. Copyright Ardor Scribendi. All rights reserved)

Cribriform trichoblastoma, also termed conventional trichoepithelioma, is a benign, well-circumscribed tumor that typically presents on the head and neck. Histologically, the cribriform variant consists of nodules or a trabecular array of basaloid cells with infundibulocystic structures. Retraction clefts separate the tumor from the rest of the dermis. No atypia, significant cellular necrosis, or atypical mitoses is seen (Figure 3) [5,6,32,35,36].

Racemiform Trichoblastoma

**Figure 4:**
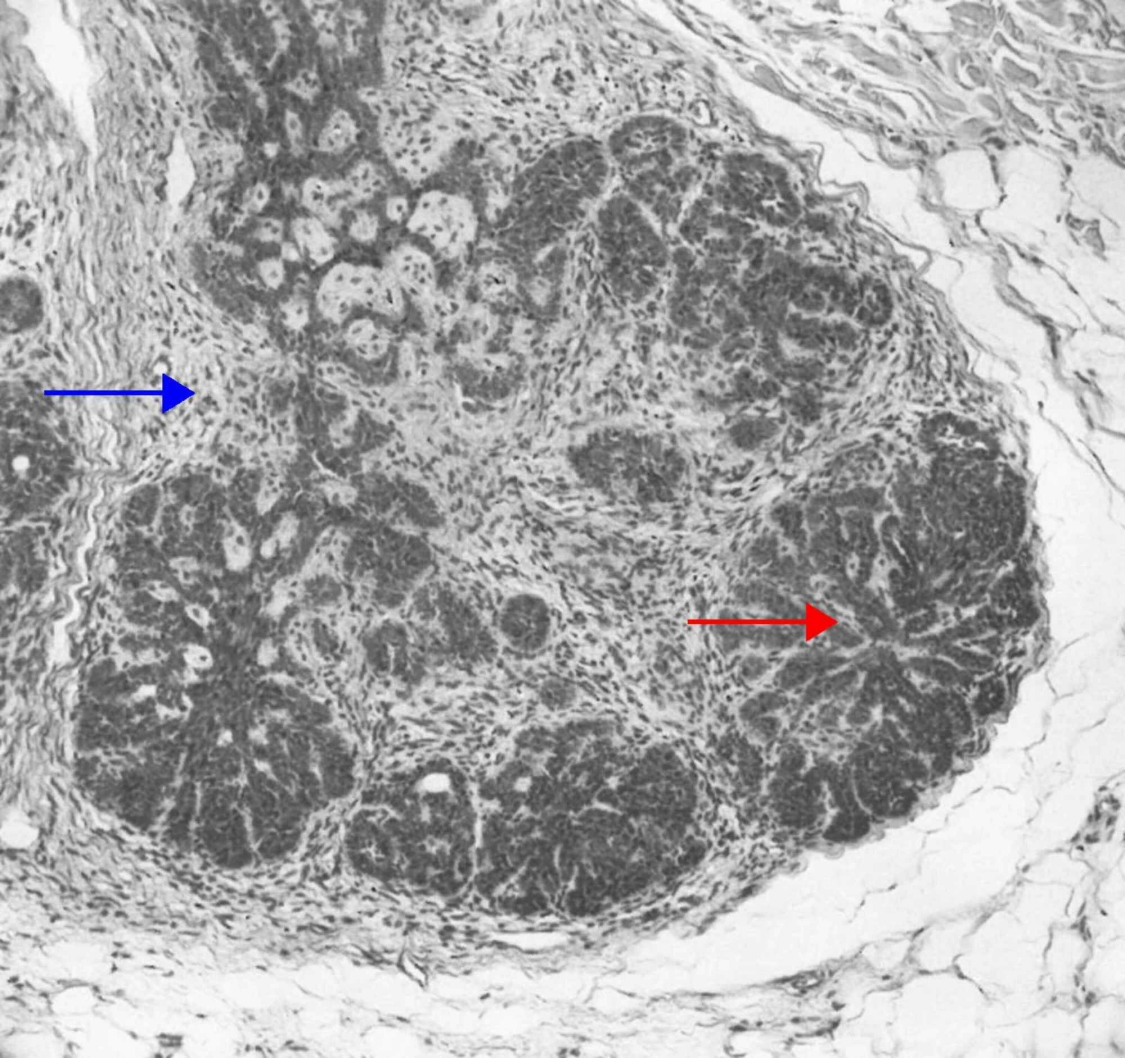
Racemiform trichoblastoma Constituted of aggregations that vaguely resemble bunches of grapes (red arrow). Although the stroma is typical of several types of trichoblastoma (e.g., small nodular, large nodular, and cribriform), that is, like embryonic perifollicular sheath (blue arrow), the germlike protuberances are not accompanied by a follicular papilla. (Adapted with permission from Ackerman A, Reddy V, Soyer H. Trichoblastoma. In: Ackerman A, Reddy V, Soyer H, editors. Neoplasms with Follicular Differentiation. New York: Ardor Scribendi; 2001. p. 588. Copyright Ardor Scribendi. All rights reserved)

Racemiform trichoblastoma is histologically characterized by epithelial nests resembling “clusters of grapes” within the stroma (Figure 4) [5,6]. Due to its histology, it is considered an unconventional trichoblastoma [32].

Columnar Trichoblastoma

**Figure 5:**
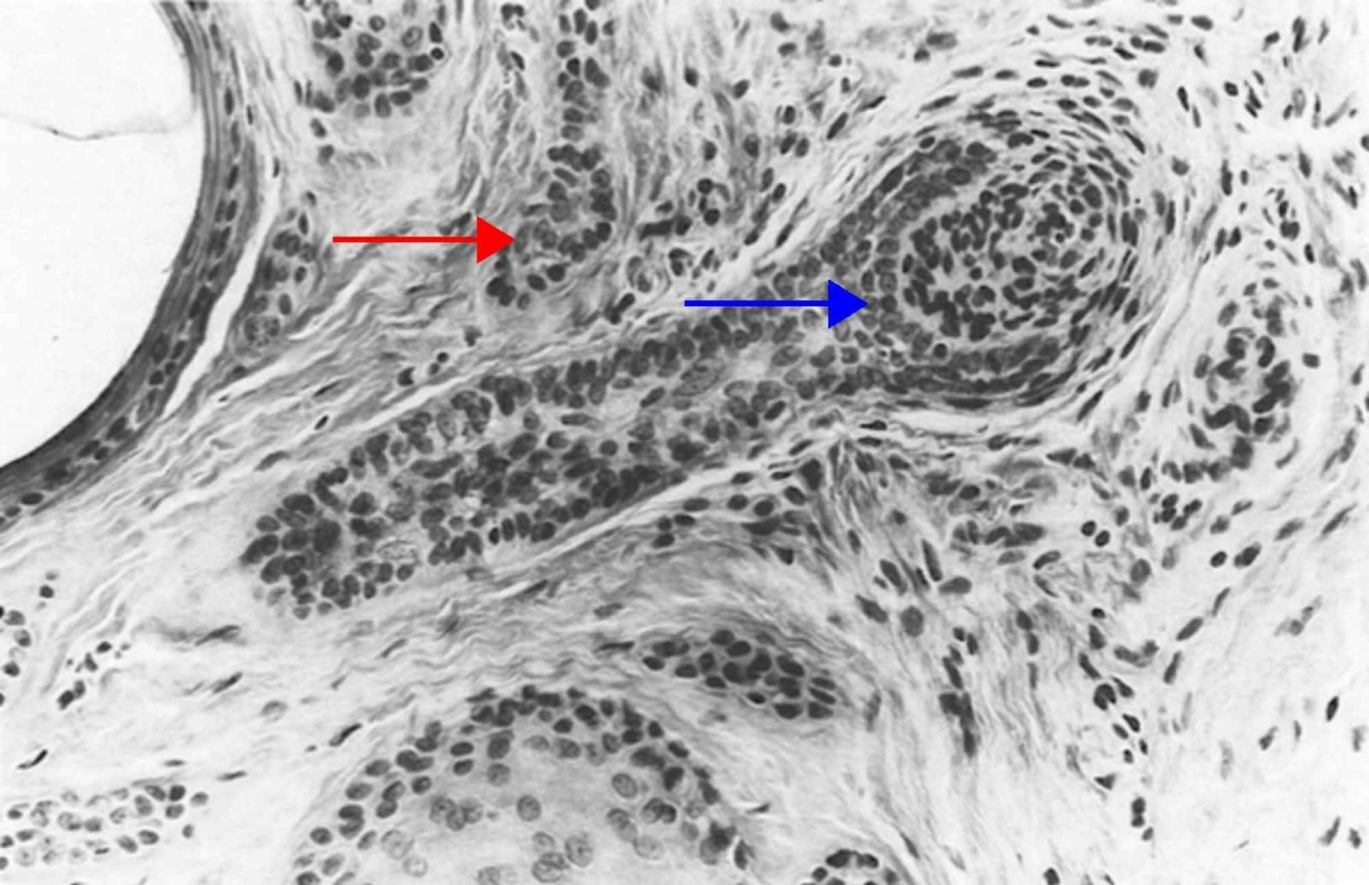
Columnar trichoblastoma (desmoplastic trichoepithelioma) May be confused with a microcystic adnexal carcinoma because of the small and large infundibulocystic structures, columns of epithelial cells that emanate from cystic structures, and depth of extension of neoplastic cells. Unlike microcystic adnexal carcinoma, however, this desmoplastic trichoepithelioma is associated with aggregations of follicular germinative cells (red arrow) and with well-defined follicular bulbs (blue arrow) and rudimentary papillae. (Adapted with permission from Ackerman A, Reddy V, Soyer H. Trichoblastoma. In: Ackerman A, Reddy V, Soyer H, editors. Neoplasms with Follicular Differentiation. New York: Ardor Scribendi; 2001. p. 601. Copyright Ardor Scribendi. All rights reserved)

Columnar trichoblastoma, also known as desmoplastic trichoepithelioma, presents as a symmetrical, well-circumscribed papule with a central depression. Histopathologically, columnar trichoblastoma is composed of narrow strands of epithelial basaloid cells, several horn cysts, granulomatous reaction, calcification, and dense fibrous stroma (Figure 5) [5,6,37,38]. The lesion is typically restricted to the papillary dermis and the upper portions of the reticular dermis [32,39].

Adamantinoid Trichoblastoma

**Figure 6:**
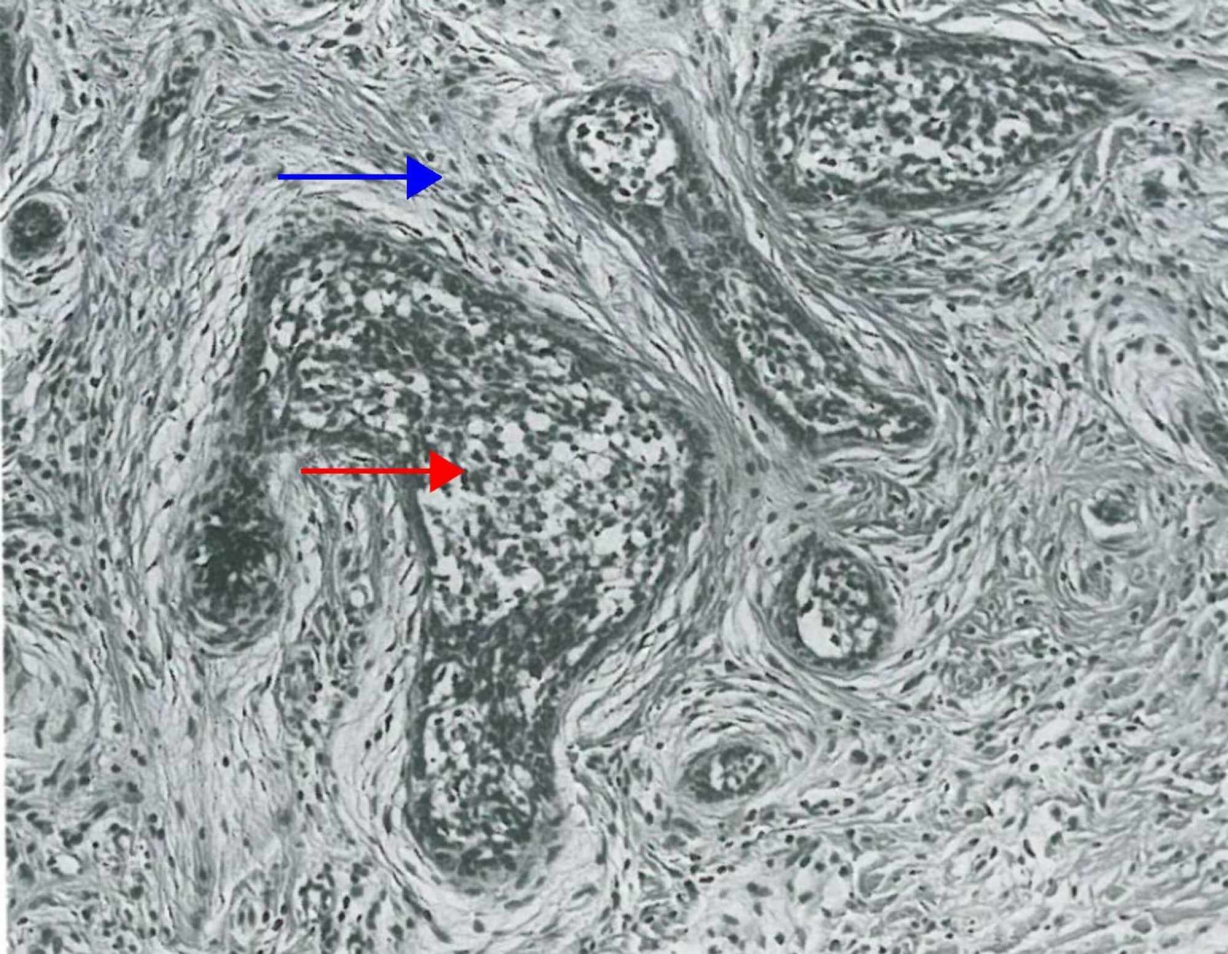
Adamantinoid trichoblastoma Aggregations of follicular germinative cells; some structures resemble follicular germs and papillae (red arrow); and pale-staining, delicate, richly fibrocystic connective tissue (blue arrow) enveloping aggregations of germinative cells simulate perifollicular sheath. The trichoblastoma is adamantinoid because the findings within aggregations closely resemble stellate reticulum of adamantinoma. (Adapted with permission from Ackerman A, Reddy V, Soyer H. Trichoblastoma. In: Ackerman A, Reddy V, Soyer H, editors. Neoplasms with Follicular Differentiation. New York: Ardor Scribendi; 2001. p. 486. Copyright Ardor Scribendi. All rights reserved)

Adamantinoid trichoblastoma, also termed cutaneous lymphadenoma, is a trichoblastoma variant that presents as a slow-growing small nodule on the face or lower limb of older adults. On histopathology, it is characterized by a well-circumscribed intradermal nodule that consists of epithelial lobules within a fibrous stroma. The lobules, though typically separate, may be interconnected. They are lined by one or more layers of small basaloid cells, with a variable peripheral palisading pattern. The tumor may expand the full thickness of the dermis or invade into the subcutis, with variable extension into the epidermis (Figure 6) [5,6,32,40].

Immunohistochemistry

Basal cell carcinoma shares similar histological features to trichoblastoma, including the presence of fissures between the epithelium and stroma, epithelial nests, and cells in a palisading arrangement on the tumor periphery. In addition to histology, immunochemistry is sometimes necessary to determine if the lesion is a basal cell carcinoma versus trichoblastoma [8]. Immunohistochemistry studies have concluded that trichoblastoma and basal cell carcinoma express similar cytokeratins, and both strongly express B-cell lymphoma 2 (Bcl-2), tumor protein p53, and the follicular differentiation markers follistatin and B-lymphoma Mo-MLV insertion region 1 (Bmi-1) [9,36,41]. However, trichoblastoma stains positive for stromal cluster of differentiation (CD) antigen 10 and CD antigen 34 as well as pleckstrin homology-like domain family A member 1 (PHLDA1), a follicular stem cell marker, whereas the basal cell carcinoma does not (Table 2) [36,42]. Nevertheless, the differentiation of an adnexal tumor from basal cell carcinoma using immunohistochemistry is somewhat controversial and is rarely performed in daily dermatopathology practice.

**Table 2:** The immunohistochemical similarities and differences between trichoblastoma and basal cell carcinoma CK5, cytokeratin 5; CK6, cytokeratin 6; CK15, cytokeratin 15; CD10, cluster of differentiation 10; CD34, cluster of differentiation 34; Bcl-2, B-cell lymphoma 2; p53, tumor protein p53; CK20, cytokeratin 20; 34BE12, cytokeratin 34 beta E12; PHLDA1, pleckstrin homology-like domain family A member 1; Bmi-1, B-lymphoma Mo-MLV insertion region 1 "+" indicates positive staining; "++" indicates strong positive staining; "-" indicates negative staining; and "+/-" indicates variable staining

	Trichoblastoma	Basal cell carcinoma
CK5, CK6, CK15 (basaloid cells)	+	+
CD10 (epithelium)	+/-	+
CD10 (stroma)	+	-
CD34 (stroma)	+	-
Bcl-2	+ peripheral	+ diffuse
p53	+	+
CK20 (epithelium)	+	-
Androgen receptor (epithelium)	-	+
34BE12	+/-	++
PHLDA1 (follicular stem cell)	+	-
Follistatin	+	+
Bmi-1	+	+

Classification

Ackerman classified hair follicle-derived adnexal cutaneous tumors into the following five subgroups based on their predominant morphological features: hair follicle and hamartomas, infundibular and isthmic tumors, tumors of the external layer, tumors originating from the matrix layer, and prominent perifollicular mesenchymal tumors [5,6]. In 2018, according to the 4th World Health Organization Classification of skin tumors, hair follicle-associated neoplasms were classified into two subgroups: benign neoplasms and malignant neoplasms (Table 3) [43]. A majority of adnexal cutaneous neoplasms are uncommon and benign [35,44].

**Table 3:** The adnexal neoplasms and neoplastic-like lesions of follicular origin

	Adnexal neoplasms and neoplastic‐like lesions
Benign	Trichoblastoma
	Trichofolliculoma
	Trichoadenoma
	Proliferating trichilemmal cyst/pilar tumor
	Trichilemmoma
	Desmoplastic trichilemmoma
	Pilomatricoma/proliferative pilomatricoma
	Infundibuloma
	Pilar sheath acanthoma
	Trichogerminoma
	Basaloid follicular hamartoma
	Basaloid epidermal proliferation overlying dermal mesenchymal lesions
Malignant	Trichilemmal carcinoma
	Trichoblastic carcinoma
	Malignant proliferating trichilemmal cyst
	Pilomatrix carcinoma

Trichofolliculoma

**Figure 7:**
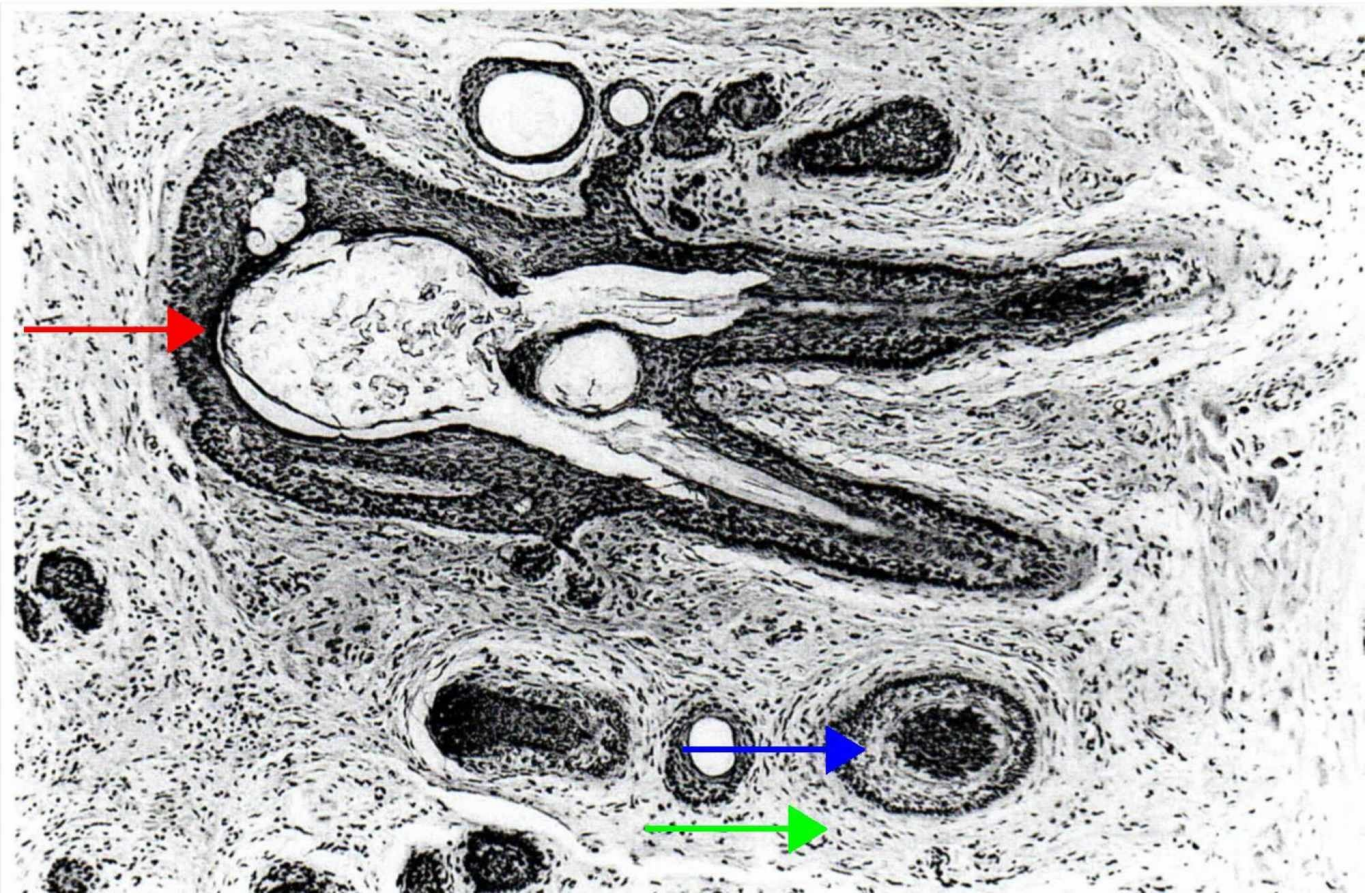
Trichofolliculoma A central infundibulocystic structure (red arrow) from which emanate smaller infundibula, from which, in turn, radiate numerous abnormal vellus follicles (blue arrow) that are surrounded by a characteristic stroma that resembles perifollicular sheath (green arrow). (Adapted with permission from Ackerman A, Reddy V, Soyer H. Trichoblastoma. In: Ackerman A, Reddy V, Soyer H, editors. Neoplasms with Follicular Differentiation. New York: Ardor Scribendi; 2001. p. 151. Copyright Ardor Scribendi. All rights reserved)

Trichofolliculoma is a benign hamartomatous lesion that presents at any age as single or multiple skin-colored nodules, with a central punctum from where the hair follicle emerges [35,44]. Histologically, trichofolliculoma is characterized by a large dilated primary follicle that is lined by infundibular stratified squamous epithelium, from which vellus hair follicles emerge (Figure 7) [5,6,45].

Trichoadenoma

**Figure 8:**
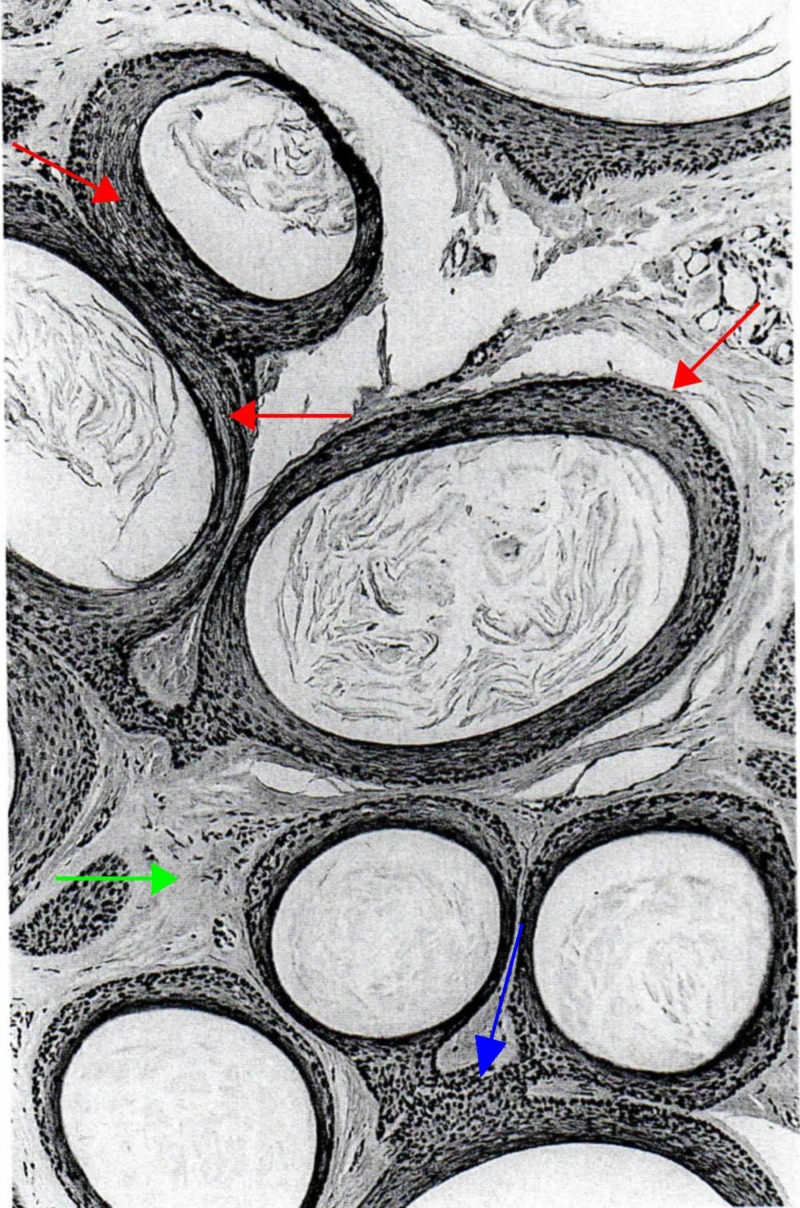
Trichoadenoma. Numerous infundibulocystic structures positioned close to one another (red arrows). Every cystic structure contains cornified cells arranged in lamellae, and are linked to one another by their lining that seems to have merged one with the other (blue arrow) or by short columns of squamous epithelium. The stroma is like that of the normal peri-infundibular dermis (green arrow), not at all like that of the perifollicular sheath. (Adapted with permission from Ackerman A, Reddy V, Soyer H. Trichoblastoma. In: Ackerman A, Reddy V, Soyer H, editors. Neoplasms with Follicular Differentiation. New York: Ardor Scribendi; 2001. p. 211. Copyright Ardor Scribendi. All rights reserved)

Trichoadenoma was first described by Nikolowski in 1958. It is a rare, benign, nodular, follicular tumor, commonly located on the face and buttocks. No gender predilection is described. Histologically, it is characterized by well-circumscribed dermal nodules with no epidermal association. The tumor is composed of many round, infundibulocystic structures separated by a fibrous stroma (Figure 8) [5,6,35,44].

Proliferating Trichilemmal Cyst

Proliferating trichilemmal cyst is a well-circumscribed, benign, solitary, nodulocystic tumor, which is often ulcerated. It ranges from 1 to 10 cm in diameter. Proliferating trichilemmal cyst has a predilection for scalps in elderly women. It can arise either de novo or from a preexisting trichilemmal cyst. Histologically, it is a well-defined solid and cystic tumor, comprised of proliferating squamous epithelium and peripherally surrounded by a hyaline membrane. It is characterized histologically by irregular anastomosing strands of keratinocytes located in the dermis and/or hypodermis [35,44].

Trichilemmoma

Trichilemmoma, also known as tricholemmoma, is a small, benign, well-circumscribed, solitary skin-colored papule that may resemble a verruca vulgaris or basal cell carcinoma. It predominantly occurs on the face in adults. These tumors originate from the outer sheath of the hair follicles. As a part of an autosomal dominant disorder, known as the Cowden’s syndrome, trichilemmomas can present as multiple oral and cutaneous lesions [35,44].

**Figure 9:**
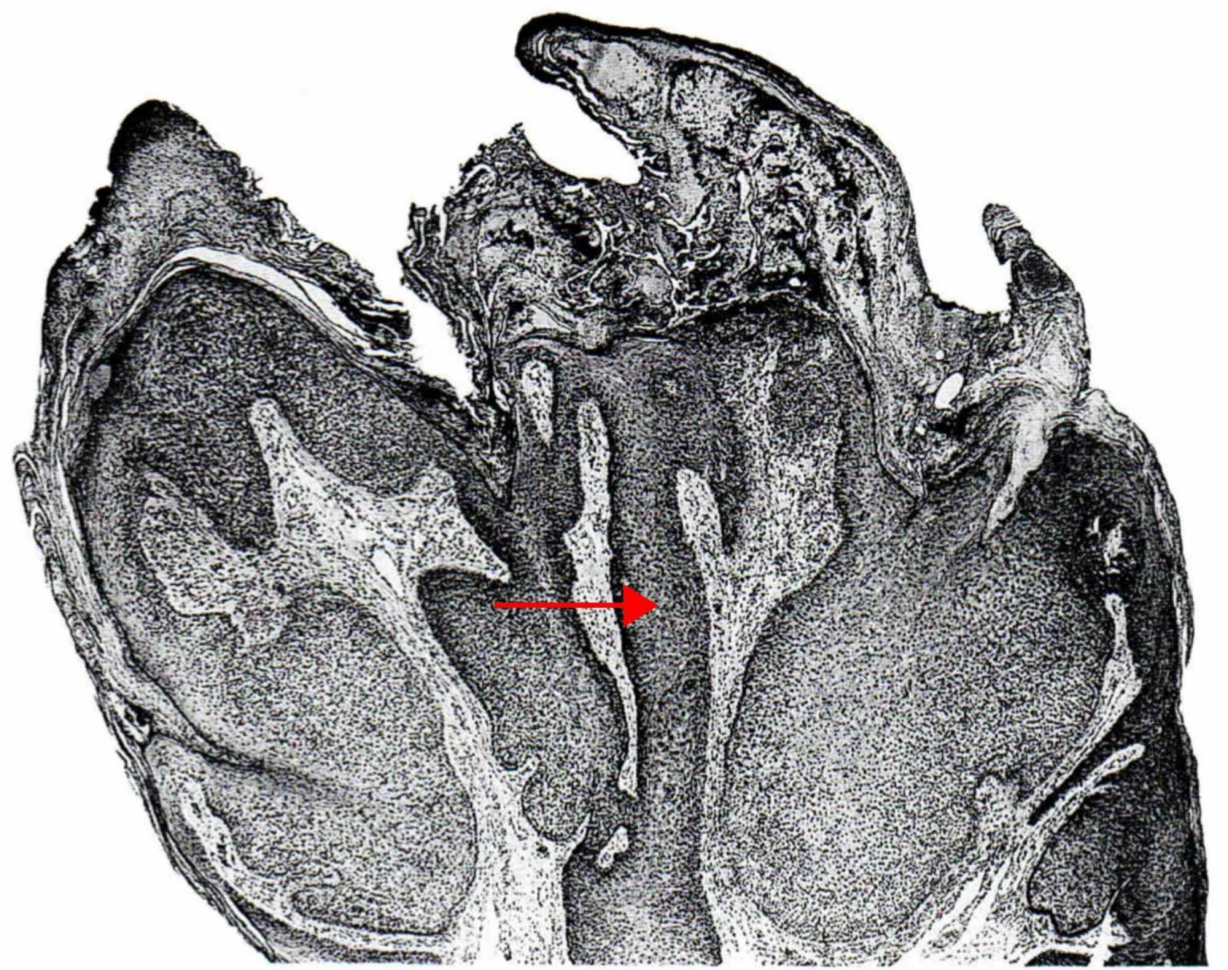
Verruca vulgaris with tricholemmal differentiation (tricholemmoma) Warts with tricholemmal differentiation (cells of hyperplastic infundibula, not neoplasia of tricholemmal cells – i.e., cells of the outer sheath) in bulbous infundibula are known conventionally as tricholemmomas, but, in reality, they are not neoplasms, but hyperplasias of infundibular epithelium (red arrow) induced by papillomavirus. (Adapted with permission from Ackerman A, Reddy V, Soyer H. Trichoblastoma. In: Ackerman A, Reddy V, Soyer H, editors. Neoplasms with Follicular Differentiation. New York: Ardor Scribendi; 2001. p. 273. Copyright Ardor Scribendi. All rights reserved)

Histologically, trichilemmoma is characterized by a well-circumscribed, papillated lesion with an exo-endophytic growth pattern (Figure 9) [5,6]. It consists of keratinocyte lobules, with cells containing a pale, glycogen-rich, clear cytoplasm located centrally, and smaller basaloid keratinocytes arranged in a palisading pattern located peripherally. The lesion is surrounded by a thick, eosinophilic hyaline basement membrane [44].

Desmoplastic Trichilemmoma

Desmoplastic trichilemmoma (DTE) is a histological variant of solitary trichilemmoma that presents as a solitary nodule and most commonly occurs on the face in young women. It is histologically characterized by strands of neoplastic cells within a dense, hypocellular, and fibrotic dermis. DTE clinically and closely resembles the morphea variant of basal cell carcinoma. However, when histologically compared to morphea basal cell carcinoma, DTE does not contain clefts between the epithelium and stroma, cellular atypia, or apoptosis [35,44].

Pilomatricoma

Pilomatricoma is a benign, solitary, firm dermal and/or subcutaneous tumor, predominantly affecting female children and adolescents, with a predilection for the head and neck area and the upper extremities. Histologically, pilomatricoma is a well-circumscribed nodulocystic tumor, predominantly located in the dermis; however, its extension into the subcutaneous tissue is not uncommon. The tumor is composed of proliferating basaloid epithelial cells with prominent nucleoli, anucleate squamous cells (“ghost cells”), eosinophilic shadow keratinized cells, and giant cell granulomas secondary to keratin clumping [35,44].

Tumor of Follicular Infundibulum

**Figure 10:**
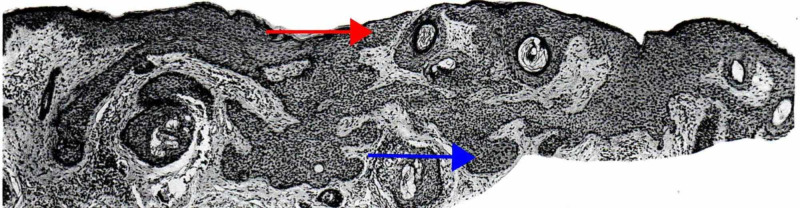
Tumor of follicular infundibulum The infundibulum and the epidermis consist of nearly identical epithelium (red arrow); not so the isthmus. The character of the cells within the anastomosing proliferation can be seen to differ from those within epidermis and infundibula. The isthmic cells that constitute tumor of follicular infundibulum (blue arrow) have less cytoplasm and less prominent intercellular bridges than those of epithelium of epidermis and infundibulum. (Adapted with permission from Ackerman A, Reddy V, Soyer H. Trichoblastoma. In: Ackerman A, Reddy V, Soyer H, editors. Neoplasms with Follicular Differentiation. New York: Ardor Scribendi; 2001. p. 317. Copyright Ardor Scribendi. All rights reserved)

Tumor of follicular infundibulum, also known as infundibuloma or isthmicoma, is a solitary, keratotic papule commonly located on the face, neck, and upper trunk in the elderly. Histologically, it is a well-circumscribed subepidermal tumor, characterized by monomorphic cells with peripherally palisading nuclei and Periodic acid-Schiff (PAS)-positive keratinocytes (Figure 10) [5,6,35,44].

Histologically, both tumor of the follicular infundibulum and trichilemmoma consist of large pale infundibular cells. However, trichilemmoma has a distinctly thickened hyaline membrane, whereas tumor of follicular infundibulum has elastic fibers instead of the hyaline membrane, surrounding the tumor periphery [35,44].

Pilar Sheath Acanthoma

**Figure 11:**
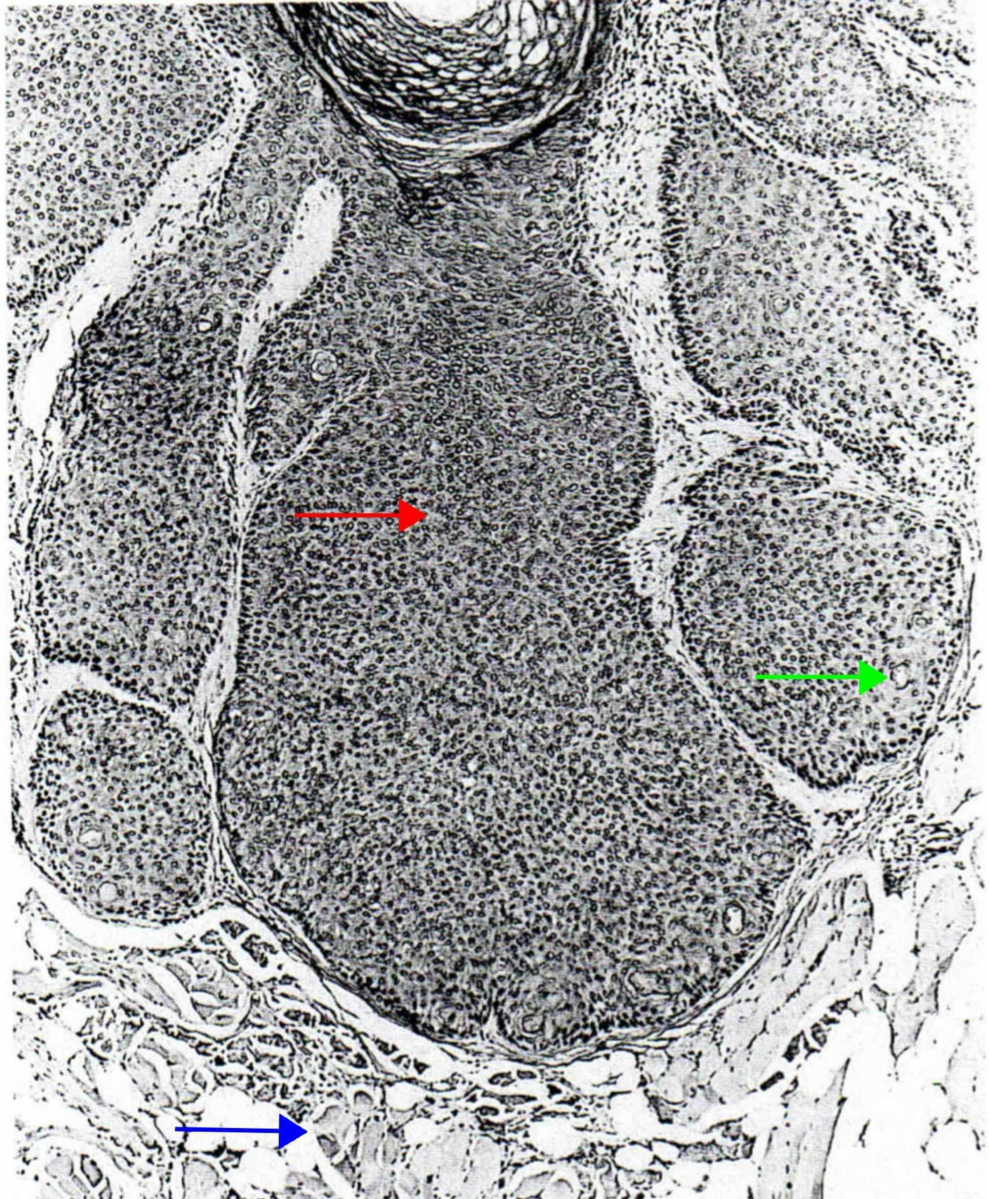
Pilar sheath acanthoma From contiguous widely dilated infundibula filled with cornified cells emerge bulbous aggregations composed of isthmic epithelium (red arrow). Those aggregations are arrayed in a radial fashion, and some extend in depth to the level of skeletal muscle (blue arrow). Sebaceous ductal structures can be seen in the aggregation of the isthmic epithelium (green arrow). Sebaceous ductal differentiation is an expected finding in pilar sheath acanthoma. (Adapted with permission from Ackerman A, Reddy V, Soyer H. Trichoblastoma. In: Ackerman A, Reddy V, Soyer H, editors. Neoplasms with Follicular Differentiation. New York: Ardor Scribendi; 2001. p. 331. Copyright Ardor Scribendi. All rights reserved)

Pilar sheath acanthoma is a rare, well-circumscribed, benign, solitary nodule with a central depression. It is typically located on the upper lip in adults. Histologically, it arises in the dermis and consists of a lobular proliferation of the benign squamous epithelium, which surrounds small cystic spaces (Figure 11) [5,6]. The tumor lacks mitosis and nuclear or cellular pleomorphism. A granular layer may be present in the surrounding layers of the tumor, along with a peripheral palisading arrangement of the cells [35,44,46].

Trichogerminoma

Trichogerminoma is a benign, well-circumscribed intradermal germinative hair follicle tumor that most commonly occurs on the head, neck, extremities, trunk, or hip. It consists of multiple lobules of basaloid cells, with a centrally located concentric arrangement of round nests along with peripheral condensation [35,44,47].

Basaloid Follicular Hamartoma

Basaloid follicular hamartoma is a rare, benign cutaneous follicular tumor that can be acquired or inherited. The lesion presents as a skin-colored papule on the face, scalp, and sometimes the trunk, most commonly affecting middle-aged and elderly individuals. It is commonly associated with basal cell carcinoma, alopecia, systemic lupus erythematosus, and myasthenia gravis. Inherited familial basaloid follicular hamartoma, an autosomal dominant disease, presents as multiple, small, flesh-colored or pigmented papules in adulthood. It may be associated with cystic fibrosis or alopecia. Histologically, basaloid follicular hamartoma consists of anastomosing squamoid and basaloid corns with intermittent horn cysts, surrounded by a loose stroma with few fibrocytes, and an absent or underdeveloped follicular papilla. Basaloid follicular hamartoma is similar to infundibulocystic basal cell carcinoma, a rare subtype of basal cell carcinoma that is superficially located, well-circumscribed, and consists of proliferated basaloid cells arranged in branching strands and cords. However, upon closer inspection, compared to basal cell carcinoma, basaloid follicular hamartoma has fewer mitoses, less cellular apoptosis, and no prolific clusters of neoplastic cells within the interfollicular dermis. Furthermore, while basaloid follicular hamartoma increases the risk for developing basal cell carcinoma, basal cell carcinoma does not directly arise from basaloid follicular hamartoma [35,44].

Basaloid Epidermal Proliferation Overlying Dermal Mesenchymal Lesions

Basaloid epidermal proliferation is a benign tumor that overlies other cutaneous mesenchymal lesions, such as dermatofibroma and cutaneous myxomas. Morphologically, while basaloid epidermal proliferation closely resembles superficial basal cell carcinoma, it can be differentiated from basal cell carcinoma by its limitation to the epidermis, lack of infiltrative growth pattern, fewer mitoses, and lack of p53 activity [35,44].

Treatment

If the diagnosis is definitively confirmed by biopsy, we recommend that the lesion be clinically monitored or surgically removed if desired. If the diagnosis is not confirmed by biopsy or the biopsy diagnosis is unclear, then another biopsy, complete surgical excision, or Mohs surgery is recommended, due to the clinical similarities between trichoblastoma and basal cell carcinoma. No trichoblastoma-specific medical or physical treatment modalities have been reported in the literature thus far [35].

Mohs and False Positive Margins

Mohs micrographic surgery is a reasonable treatment for trichoblastoma when the lesion (1) occurs on the face, genitals, hands, or feet, (2) is large and aggressive, or (3) is histologically indistinguishable from basal cell carcinoma and meets the Mohs micrographic surgery criteria for basal cell carcinoma. While Mohs micrographic surgery tends to provide the lowest recurrence rates for trichoblastoma, recurrence may be observed due to false-negative tumor-free margins, commonly as a result of the multi-focality folliculocentric basaloid proliferations in the fibrotic mesenchymal stroma [21].

In the literature, only five cases have reported using Mohs micrographic surgery as a treatment modality for trichoblastoma. Of these, four utilized Mohs micrographic surgery due to the location of the tumor on the face: two on the nasal ala, one on the eyelid, and one on the nasal cheek fold [29,48,49]. Two of these cases resulted in recurrence, due to false-negative tumor-free margins. In the fifth case, Mohs micrographic surgery was preferred due to the size of the lesion and its aggressive growth pattern [48].

Prognosis

In most cases, trichoblastoma has a favorable prognosis. A review of existing literature reveals low incidences of recurrence, progression, or association with malignancy [23]. The progression or association with malignancy tends to be reported when trichoblastoma has recurred or has been present for more than five years [50].

## Conclusions

Trichoblastoma is a benign tumor arising from an aberrant proliferation of primitive follicular germinate cells. Clinically, it can be challenging to differentiate trichoblastoma from basal cell carcinoma even with the aid of dermoscopy. Histology and immunohistochemistry are typically needed to accurately identify trichoblastoma. Trichoblastoma typically has a favorable prognosis, with a low incidence of progression, recurrence, or association with malignancy. With the correct diagnosis in hand, malignancy can be ruled out, and the best treatment and prognosis can be provided.

## References

[1] Zeller KA, Billmire DF (2012). Trichoblastoma: management of a rare skin lesion. J Pediatr Surg.

[2] Pitarch G, Botella-Estrada R (2015). Dermoscopic findings in trichoblastoma. Actas Dermosifiliogr.

[3] Ghigliotti G, De Col E, Parodi A, Bombonato C, Argenziano G (2016). Trichoblastoma: is a clinical or dermoscopic diagnosis possible?. J Eur Acad Dermatol Venereol.

[4] Headington JT (1970). Differentiating neoplasm of hair germ. J Clin Pathol.

[5] Ackerman A, de Viragh P, Chongchitnant N (1993). Trichoblastoma. Neoplasms with Follicular Differentiation.

[6] Ackerman A, Reddy V, Soyer H (2001). Trichoblastoma. Neoplasms with Follicular Differentiation.

[7] Jacob A (1827). Observations respecting an ulcer of peculiar character, which attacks the eyelids and other parts of the face. Dublin Hospital Rep Commun Med Surg.

[8] Vega Memije ME, Luna EM, de Almeida OP, Taylor AM, Cuevas Gonzalez JC (2014). Immunohistochemistry panel for differential diagnosis of basal cell carcinoma and trichoblastoma. Int J Trichology.

[9] Kim HC, Sohng SH, Shin DH, Choi JS, Bae YK (2016). Immunohistochemical expression of cytokeratin 15, cytokeratin 19, follistatin, and Bmi-1 in basal cell carcinoma. Int J Dermatol.

[10] Wu S, Han J, Li WQ, Li T, Qureshi AA (2013). Basal-cell carcinoma incidence and associated risk factors in U.S. women and men. Am J Epidemiol.

[11] Hannuksela-Svahn A, Pukkala E, Karvonen J (1999). Basal cell skin carcinoma and other nonmelanoma skin cancers in Finland from 1956 through 1995. Arch Dermatol.

[12] Green A, Battistutta D, Hart V, Leslie D, Weedon D (1996). Skin cancer in a subtropical Australian population: incidence and lack of association with occupation. Am J Epidemiol.

[13] Chuang TY, Popescu A, Su WP, Chute CG (1990). Basal cell carcinoma. A population-based incidence study in Rochester, Minnesota. J Am Acad Dermatol.

[14] Mackiewicz-Wysocka M, Bowszyc-Dmochowska M, Strzelecka-Weklar D, Danczak-Pazdrowska A, Adamski Z (2013). Basal cell carcinoma - diagnosis. Contemp Oncol.

[15] Loh SH, Lew BL, Sim WY (2015). Composite tumor associating Trichoblastoma and Seborrheic Keratosis. Ann Dermatol.

[16] Rofagha R, Usmani AS, Vadmal M, Hessel AB, Pellegrini AE (2001). Trichoblastic carcinoma: a report of two cases of a deeply infiltrative trichoblastic neoplasm. Dermatol Surg.

[17] Mencia-Gutierrez E, Gutierrez-Diaz E, Ricoy JR, Rodriguez-Peralto JL (2003). Eyelid trichoblastoma: an unusual localization. Int J Dermatol.

[18] Kaddu S, Schaeppi H, Kerl H, Soyer HP (1999). Subcutaneous trichoblastoma. J Cutan Pathol.

[19] Graham BS, Barr RJ (2000). Rippled-pattern sebaceous trichoblastoma. J Cutan Pathol.

[20] Swick BL, Baum CL, Walling HW (2009). Rippled-pattern trichoblastoma with apocrine differentiation arising in a nevus sebaceus: report of a case and review of the literature. J Cutan Pathol.

[21] Umbert P, Munoz JF (2012). False-negative tumor-free margins following mohs surgery for aggressive trichoblastoma. Am J Dermatopathol.

[22] Wang YJ, Tang TY, Wang JY, Huang YK, Wu YH (2018). Genital basal cell carcinoma, a different pathogenesis from sun-exposed basal cell carcinoma? A case-control study of 30 cases. J Cutan Pathol.

[23] Regauer S, Beham-Schmid C, Okcu M, Hartner E, Mannweiler S (2000). Trichoblastic carcinoma ("malignant trichoblastoma") with lymphatic and hematogenous metastases. Mod Pathol.

[24] Pellegrini C, Maturo MG, Di Nardo L, Ciciarelli V, Gutierrez Garcia-Rodrigo C, Fargnoli MC (2017). Understanding the molecular genetics of basal cell carcinoma. Int J Mol Sci.

[25] Epstein EH (2008). Basal cell carcinomas: attack of the hedgehog. Nat Rev Cancer.

[26] Otsuka A, Levesque MP, Dummer R, Kabashima K (2015). Hedgehog signaling in basal cell carcinoma. J Dermatol Sci.

[27] Xie P, Lefrancois P (2018). Efficacy, safety, and comparison of sonic hedgehog inhibitors in basal cell carcinomas: a systematic review and meta-analysis. J Am Acad Dermatol.

[28] Basic-Jukic N (2017). Trichoblastoma cutis in a renal transplant recipient: a case report. Transplant Proc.

[29] Johnson TV, Wojno TH, Grossniklaus HE (2011). Trichoblastoma of the eyelid. Ophthalmic Plast Reconstr Surg.

[30] Kang TW, Kang H, Kim HO, Song KY, Park YM (2009). Trichoblastoma in a child. Pediatr Dermatol.

[31] Dileonardo M (1995). Trichoblastomas: small nodular vs. large nodular vs. cribriform. Dermatopathol Pract Concept.

[32] Kaddu S (2011). Tumours with Follicular Differentiation. Skin Cancer - A World-Wide Perspective.

[33] Teli B, Thrishuli PB, Santhosh R, Amar DN, Rajpurohit S (2015). Giant solitary trichoepithelioma. South Asian J Cancer.

[34] Genc S, Sirin Ugur S, Arslan IB (2012). A giant solitary trichoepithelioma originating from the auricle. Dermatol Surg.

[35] Tellechea O, Cardoso JC, Reis JP, Ramos L, Gameiro AR, Coutinho I, Baptista AP (2015). Benign follicular tumors. An Bras Dermatol.

[36] Stanoszek LM, Wang GY, Harms PW (2017). Histologic mimics of basal cell carcinoma. Arch Pathol Lab Med.

[37] Wada M, Hanada K, Kanehisa F, Asai J, Takenaka H, Katoh N (2013). A case of desmoplastic trichoepithelioma with ossification. Indian J Dermatol.

[38] Ardigo M, Zieff J, Scope A, Gill M, Spencer P, Deng L, Marghoob AA (2007). Dermoscopic and reflectance confocal microscope findings of trichoepithelioma. Dermatol.

[39] Wang Q, Ghimire D, Wang J (2015). Desmoplastic trichoepithelioma: a clinicopathological study of three cases and a review of the literature. Oncol Lett.

[40] Yu R, Salama S, Alowami S (2014). Cutaneous lymphadenoma: a rare case and brief review of a diagnostic pitfall. Rare tumors.

[41] Cordoba A, Guerrero D, Larrinaga B, Iglesias ME, Arrechea MA, Yanguas JI (2009). Bcl-2 and CD10 expression in the differential diagnosis of trichoblastoma, basal cell carcinoma, and basal cell carcinoma with follicular differentiation. Int J Dermatol.

[42] Battistella M, Peltre B, Cribier B (2014). PHLDA1, a follicular stem cell marker, differentiates clear-cell/granular-cell trichoblastoma and clear-cell/granular cell basal cell carcinoma: a case-control study, with first description of granular-cell trichoblastoma. Am J Dermatopathol.

[43] Elder DE, Massi D, Scolyer RA, Willemze R (2018). WHO Classification of Skin Tumours.

[44] Alsaad KO, Obaidat NA, Ghazarian D (2007). Skin adnexal neoplasms--part 1: an approach to tumours of the pilosebaceous unit. J Clin Pathol.

[45] Plewig G (1980). Sebaceous trichofolliculoma. J Cutan Pathol.

[46] Baja A (2019). Follicular, infundibular, hamartoma-pilar sheath acanthoma. Int J cell Sci Mol Biol.

[47] Chen LL, Hu JT, Li Y (2013). Trichogerminoma, a rare cutaneous follicular neoplasm with indolent clinical course: report of two cases and review of literature. Diagn Pathol.

[48] Cowen EW, Helm KF, Billingsley EM (2000). An unusually aggressive trichoblastoma. J Am Acad Dermatol.

[49] Pontes LT, Simiao AL, Seugling FdR, Stelini RF, Cintra ML, de Souza EM, Moraes AM (2012). Mohs micrographic surgery on recurrent trichoblastoma. Surg Cosmet Dermatol.

[50] Nguyen LV, Masouminia M, Choy JO, Peng SK, Ji P, French SW (2017). Atypical giant trichoblastoma: an unusual presentation. Exp Mol Pathol.

